# Navigating work, family, and society: Challenges facing Jordanian female journalists

**DOI:** 10.1371/journal.pone.0343919

**Published:** 2026-03-02

**Authors:** Hana Al-Souob, Enas A Assaf

**Affiliations:** 1 Radio and Television Department, Faculty of Mass Communication, University of Petra, Amman- Jordan; 2 College of Nursing, King Khaled University, Khamis Mushait, Saudi Arabia; Shandong University, CHINA

## Abstract

The aim of this study is to explore the challenges encountered by Jordanian female journalists, focusing on their experiences in the journalistic work environment (including the workplace and the field) in addition to the challenges faced from society and family. Through using a qualitative and inductive approach by conducting in-depth interviews with Jordanian female journalists who are registered professionals working in a variety of media environments, including daily, weekly, and electronic media. The research findings explore a wide range of challenges female journalists face in Jordan, including workplace issues and external challenges from family and society. The following main themes were emerged in the study, at the workplace level: harassment and newsroom safety; pay inequalities, organizational culture and leadership this point leads to subthemes as bullying, framing female Journalists, and stigmatization, On the other hands challenges themes found at the level of the society and family security which emerged several subthemes as party affiliation, security harassment, and cultural beliefs, as well as family, parents and relatives perspectives. The findings highlight persistent gender‑based discrimination shaped by workplace practices and cultural expectations. Strengthening protections, ensuring fair pay and promotion, and shifting societal attitudes are essential to supporting female journalists. Further research is needed to better understand these challenges and inform effective solutions.

## Introduction

Female journalists have played a crucial role in shaping and advancing the media industry and have contributed significantly to their development. Their noticeable viewpoints contribute to diverse and inclusive news coverage, creating a richer and more representative media landscape. However, a global report from the International Media for Women’s Foundation stated that only 35.1% of journalists were women [1]. Many challenges have been reported among female journalists worldwide in the work environment, including unreasonable treatment, gender discrimination, recognized stereotyped attitudes, sexual harassment by male colleagues, and job insecurity; at the social level, there is a lack of social support and socially unacceptable attitudes [2–7].

The trajectory of Arab females, specifically Jordanian women, in the workforce has undergone significant transformations marked by distinct stages of change and development. In the early days of Jordanian society (patriarchy and tribal society), women primarily engaged in simple home-based activities and small handicrafts. Over time, women expanded their roles in the realm of education, health, industry, the economy and trade, public relations, and office administration [8]. However, the field of media has posed unique challenges, with social barriers impeding women’s participation. Despite these challenges, the presence of creative and accomplished females in journalism, radio, television, and electronic media, as documented by Haddadin [9], signifies resilience and showcases the potential to break down societal norms and foster a more inclusive media landscape.

### Jordanian women and journalism

The emergence of journalism in Jordan was closely linked to the arrival of the founding King Abdullah I Ibn Al-Hussein to Ma’an, and to the establishment of the Emirate of Transjordan, when the founding king decided to create a newspaper called “Al-Haqq Ya’alou,” to be launched from the city of Ma’an. It began publishing in 1920, and it was the first newspaper published in Jordan.

The fifties era is the real starting point for the modern Jordanian press, because the Jordan Press Association Law was issued for the first time in 1953.

The sixties and seventies were characterized by the issuance of relatively large numbers of daily and weekly newspapers, most notably “Al-Dustour” newspaper in 1967. Then, “Al-Rai” newspaper in 1971. In 1969, the Jordanian News Agency (Petra) was established. On the other hand, the media sector has developed over the past twenty years when Jordan witnessed a remarkable growth in the number of newspaper and electronic news websites (19 Print Press Publications,133 Electronic Publications), and Radio & Television stations also spread clearly during this period (19 TV stations, 41 Radio stations) [10]. Although Jordanian women entered the world of journalism early and succeeded in covering many major political events and complex social issues, their participation in drawing up media policies and managing media institutions is limited compared to men, even though she assumed the position of Minister of Information in 1984 and a government spokesperson in 2003.

During the past twenty years, several women have headed major journalistic institutions, such as the English daily Jordan Times, Al-Ghad newspaper, the Radio and Television Corporation, and the Jordan News Agency. In the Jordanian Press Association, women registered a presence in the Association at a rate of 20%, and the percentage of women reaching leadership positions did not exceed 2% [11].

### Challenges of female journalists

The obstacles that hinder Arab females’ moving up to decision-making positions in media are attributed to three major factors: societal, institutional, and journalists themselves [12]. In many respects, the challenges faced by Jordanian female journalists in the media work environment have become threatening to the continuation of female journalists in the profession. However, the challenges faced by female journalists seem to affect their work satisfaction, as was found in one study in Jordan [13] and others in Palestine [14], which in overall might lead to leaving the media workforce, as what Ananzeh [15] found. On the other hand, Inequality in pay remains a persistent problem globally, as women in all regions receive lower pay than men, and the gender-based pay gap is estimated at 23% globally according to the international labor law and organization [16,17], and at about 20% according to United Nation and Forum WE [18,19]. Although in 1966 Jordan ratified ILO Convention No. 100 of 1951 on Equal Remuneration, and in 1963 it ratified ILO Convention No. 111 of 1958 on Discrimination (in Employment and Occupation). However, the provisions of these agreements have not yet been reflected in Jordanian law [20].

It is clear that Jordanian legislation does not include provisions related to the principle of equal pay for male and female workers when working is equal [21]. The Labor Law does not explicitly prohibit discrimination at work, i.e., the right of men and women to equal pay, and no party is punished for failing to adhere to the principle of equal pay [20].

In 2020, the World Bank estimated the pay gap between male and female workers in the Jordanian private sector at about 17%, despite the similar professions, experience, and educational attainment of these women and men [17].

Given the gaps highlighted in prior studies on female journalist’s challenges, this study draws upon established theoretical perspectives to provide a stronger conceptual foundation. This study is referred to as an umbrella to gender theories: biological theory, social construction theory, functional theory, and socialization theory [22]. These frameworks provide a focused and clear understanding of how gendered expectations, cultural norms, overlapping identities, and media structures shape the experiences of women in journalism. These theories helped in the study design ensures that the research questions and subsequent analysis are not only informed by prior scholarship but also situated within broader conceptual debates on gender and media [23]. This theoretical follow can strengthen the connection between the literature review and the study findings, allowing the study to contribute both to practical view and the theoretical discussions in media and communication studies.

Although the growing body of literature on gender dynamics in Jordanian workplaces, prior studies have predominantly focused on some of the challenges faced by women in the broader labor force, such as workplace discrimination and access to employment. However, these investigations have largely overlooked the multifaceted barriers inherent to journalism—a profession that demands not only professional resilience but also robust collective support from family and community structures, which are deeply intertwined with Jordan’s cultural and social norms. Therefore, the aim of this study is to explore the challenges encountered by Jordanian female journalists, focusing on their experiences in the journalistic work environment (including the workplace and the field) in addition to the challenges faced from society and family.

### Study questions

The main question of this study is:

What are the challenges faced by Jordanian female journalists and their impact on the status of their professional practice and personal life?

Which gives rise to the following sub-questions:

1. What challenges do Jordanian female journalists face in the workplace within press institutions?
2. What challenges do Jordanian female journalists face in field journalism outside of press institutions?
3. What challenges do Jordanian female journalists face from family?
4. What challenges do Jordanian female journalists face from society and the cultural perspectives?

## Materials and methods

### Study design

This study is a qualitative study design and was based on one-to-one interviews. The interviews were conducted and transcribed in Arabic, which is the official language of the country. All quotes included in this manuscript were translated and back-translated by two members of the research team who are bilingual. Due to financial limitations, only quotes included in this manuscript were translated into English. The gender theories were used as a guide for the study method and analysis of the study.

The study depends on the following two approaches to reach its results. First, the descriptive qualitative approach, by conducting in-depth interviews with Jordanian female journalists who are registered as members of the Jordanian Press Association to analyze their experiences and the challenges they face in terms of organization and field of journalism, and on the family and society front. According to Hesse-Biber [24], qualitative research seeks depth in data and its analysis rather than pursuing the quantitative dimension. It is the best tool for enhancing and refining data in a manner that clearly exposes the basic features of the studied cases. The second approach: is the inductive approach, which proceeds from the specific to the general to reach conclusions based on observation and experimentation to arrive at a law or general rule. The researchers performed this by analytically observing the parts and sub-points related to the research subject by generalizing the results arrived at after testing some of the parts and cases, and then by generalizing the results to all parts or cases that form the subject of the research. Wonjnar & Swans [25] present that this approach depends on the study of the parts up to the overall issues and that govern the phenomenon by monitoring or observing some individual cases that lead a person to hypotheses to be verified up to the general description that encompasses all the parts and sub-points of the phenomenon.

### Study participants

The list of the Jordanian Press Association at the time of the study indicated that there were 1,310 registered journalists. The study community consisted of Jordanian female journalists registered as members of the Jordanian Press Association in the Hashemite Kingdom of Jordan who are currently working.

The study adopted the purposive sample that consisted of 134 Jordanian female journalists registered with the Jordanian Press Association and working in daily and weekly newspapers, electronic news websites, and the Jordan News Agency “Petra”. The inclusion criteria were: participants must be members of the Jordanian Press Association., must be currently employed and work in person, not remotely for either the Jordan News Agency “Petra”, a daily or weekly newspaper, or a news website licensed by the Jordan Media Commission, and must agree to conduct the interview and publish its content for research purposes. Of these, 15 female journalists participated in semi-structured interviews form different agencies. This took place until the study reached the level of theoretical saturation, and the interviews no longer added new information [24].

The researchers conducted 15 semi-structured interviews on both public and private Press institutions with female journalists (n = 9) and female editors-in-chief (n = 6). Six worked for daily newspapers, three for weekly newspapers, two for Jordanian news agencies, and four for electronic news websites. Female journalists work in different press institutions, which helped form a clearer picture of the challenges facing these female journalists.

Their average age was 36 years; six of them were married, and the average duration of their employment in journalism was 13 years. Regarding educational level, 14 of them obtained a bachelor’s degree and only one obtained a diploma. The researchers conducted all interviews.

To preserve anonymity, the researchers removed all identifying names of the individuals and press institutions for which they worked when presenting the interview data.

### Data collection procedure

Participants were approached based on their eligibility according to the criteria of the study. Once they verbally agreed to participate, they were provided with an information sheet detailing the study’s aim, process, and right to withdraw at any time. Participants were then asked to sign their consent to participate in the study. Participants were also informed that the interview location and schedule were selected based on their preferences.

The interviews lasted on average from 45–60 minutes. The authors both conducted the interviews. The first two interviews were recorded after obtaining the journalists’ consent to be recorded. These interviews were then transcribed on paper. However, since the authors felt that recording might limit the journalists’ ability to express their feelings freely, even to the point of tears, they decided to carefully transcribe all answers on paper so that the journalists could respond with ease. Before the end of each interview, the authors reviewed all answers with each interviewee to ensure that the authors had accurately transcribed their notes.

The data collection was continued until data saturation was reached. The interviews were conducted between May and December 2023. All the data were kept within a locked cabinet, and only the researchers had access to this cabinet.

### Data analysis

The authors analysed the interview data using content analysis, following the seven steps outlined by Colaizzi [26]. First, the authors immersed themselves in the participants’ comments by thoroughly reading and rereading the interviews to identify significant statements related to the phenomenon. These statements were coded and organized by the two authors independently. Next, each significant statement was categorized into themes after discussion among the two authors. A comprehensive descriptive analysis was developed by integrating the findings of these themes. The fourth step involves formulating detailed descriptions that capture the essential qualities of the phenomenon. These descriptions were then reviewed and validated in the fifth step by comparing them with those of the original interviews. Finally, the findings were presented in a comprehensive report that accurately reflected participants’ experiences. A validation process was conducted to ensure the accuracy of the descriptive phenomenological analysis. Four randomly selected participants were contacted, and suitable calls were arranged. This approach protected the participants’ physical and psychological safety during the validation process. The integrated findings were either validated or adjusted based on the participants’ final confirmation or revisions to the description of their comments.

### Ethical consideration

The University of Petra approved the study (IRB No. S/2b/4/2023). All participants were required to sign a consent form before taking part in the study. Throughout the research process, the privacy, anonymity, and autonomy of each study participant were upheld. Participation was voluntary and they could withdraw from the study without explanation.

### Findings

The following section presents the findings, which are organized around the workplace level: harassment and newsroom safety; pay inequalities, organizational culture and leadership this point leads to subthemes as bullying, framing female Journalists, and discrimination. On the other hands challenges themes found at the level of the society and family security which emerged several subthemes as party affiliation, security harassment, and cultural beliefs, as well as family, parents and relatives perspectives as well as the cultural perspectives. These themes collectively highlight the multifaceted challenges faced by female journalists.

### Workplace challenges

#### Harassment and newsroom safety.

The interviewees’ responses differed on the issue of the existence of verbal or sexual harassment inside work institutions, but there was semi-agreement that news websites have more reported harassment than printed newspapers. As highlighted by Participant No. 3 as she said that “sexual harassment does not exist in large journalistic institutions”; an opinion that was challenged by Participant No. 9, who supported the presence of harassment in all work environments, but the issue – according to her – depends on the girl’s personality, strength, and ability to restrain the other party. Participants described certain forms of verbal harassment that mirrored expressions commonly used in street language. For example, Participants No. 3 and No. 10 reported hearing the phrase, “What is this surface-to-air rocket?” In colloquial slang, the term “rocket” is used to describe a very beautiful girl, highlighting how everyday language was employed in a derogatory manner within professional settings.

While, on very few news sites, they try to exploit the femininity and beauty of female media professionals to solicit advertising contracts from certain institutions; however, this was mentioned only by two participants. Participant No. 8 said that sometimes the female media professional is asked to “meet so-and-so, fix your appearance, and wear short clothes.” Participant No. 12 referred to this matter and said, some of those in charge of certain news sites do not observe professional ethics and ask female media professionals to bring advertisements in any way it takes. This was mentioned by Participant No. 8 as the existence of types of harassment, some of which take the side of flirting, such as *“Your voice is sweet,”* and sometimes develop into insults, especially when the female media professional rejects such practices. And mentioned also that the matter is not limited to words, as there are sometimes indecent questions that in certain cases infringes on the most subtle private concerns of the female media professional.

In the course of fields of journalists for example, covering field events such as demonstrations and protests, some physical harassers could be justified by blaming female journalists. Participant No. 8 refers to by sentences and phrases they use, such as: *“It is your fault … why are forcing yourself with the crowds?”.*.

#### Pay and promotion inequities.

Pay and promotion inequalities, were found through discussions and interviews with the participants that there is a wage gap between female and male journalists that leans in favour of males on electronic news websites, and to a lesser extent in daily newspapers.

Participant No. 10 indicated that there were inconsistencies and confusion regarding salaries on electronic news websites. Usually, males receive much higher salaries than do female journalists, even though their work is the same. Participant No. 11 pointed out that the relationship between journalists and the director was the reason for the wage gap.

Regarding bonuses and promotions, most participants indicated that they were directly related to the issue of proximity to the person in charge, or having common interests or personal relationships. This is present in both types of papers and electronic press institutions.

The participants’ answers indicated that there was a discrepancy also in bonuses and promotions between male and female journalists in favour of males. This feeling of discrimination existed among the majority of participants (12 out of 15).

The absence of a fixed salary scale that is equal between the genders when determining the salary of appointment, rewards and incentives in press institutions, as well as the link between setting the salary and the mood of the owner or editor-in-chief of news websites, contributes significantly to deepening the pay gap between male and female journalists, which leaves negative economic, social and psychological effects on female journalists because they perform the same work of equal value with the work provided by male journalists and perhaps with better quality.

Regarding promotions, the disparity is more pronounced in digital news sites than in printed newspapers. Printed newspapers typically follow a career ladder structure, benefitting from their long history. However, the established traditions and practices in promotions have not yet achieved full justices, based on the majority of female participants’ opinions (10 out of 15).

### Organizational culture and leadership

Most participants noted various forms of **bullying**, starting with the stigmatization of female journalists by portraying them as lacking competence and efficiency. This can extend to linking these issues to the natural aspects of their bodies, disregarding their achievements and ability to meet the goals of newspapers or websites. Participant No. 13 specifically mentioned the ridicule of female journalists’ achievements, labeling them backward and incapable of accessing information. The disturbing aspect is the demeaning remark that, in the end, “she remains a woman,” as if being a woman is considered an insult. Strikingly, female journalists have approved such statements.

On the other hand**, framing female journalists and stigmatizing** them with certain characteristics. In that, some participants indicated that male colleagues in the media stigmatized females and framed their success or movement with negative connotations aimed at securing success and achievements. Participant No. 3 referred to this when she said, some colleagues denounce females’ assumption of an administrative position in the press institution’. In the same context, Participant No. 11 and 14 indicated that the male media person is generally sensitive to the success of his female colleague, looks at her with suspicion, wants to take his acquired rights, and treats her as if she were a foreigner or an expatriate worker. The participant added that the editor-in-chief of her newspaper joked with her once and said: “He will suggest that the government issues a work permit for females, such as those issued to non-Jordanian workers.”

Other challenge emerged in the organizational culture is **discrimination within the press institution**, by which, the vast majority of the interviewees (13 out of 15) indicated that there are many forms of discrimination against them in their journalistic work, and these forms vary between salary gaps, promotions, travel, assignments, treatment, stigmatization, framing, and other issues. Participant No. 7 said, ‘Due to the orientations of the Islamic newspaper, we, as females, had a private room, and this I considered positive from the point of view of maintaining tranquility and the privacy of females, but from another angle it can be perceived as being negative, because it may reduce the status of females.

There are other forms of discrimination, such as **the distribution of assignments**. There are self-governing sovereign sectors that are covered by male journalists only, and administrations in both printed newspapers and electronic news websites that are deliberately assigned to men only, which could be based on the patriarchal society image. Participant No. 10 emphasized this issue and said: “When I asked to be assigned to cover the news of a sovereign institution, the editor-in-chief told me directly and frankly that you are a woman, and he did not explain to me other justifications, as if the assumption is that the word woman means lower status”. Participant No. 7 fully agreed with this comment.

As for the **tribal issue**, there is discrimination based on the name of the tribe, so the matter is reflected in printed newspapers and electronic news websites because some ministries and institutions are not enthusiastic about giving information unless the male or female journalist is the son or daughter of a tribe. Electronic news websites intentionally resort to appointing journalists from tribal members so that they can obtain information from sources easily. They also resort to writing the name of the journalist on the published press material, because the name of the journalist’s clan gives the published material greater power. This was supported by Participants No. 8 and 14.

When considering leadership positions, if a female journalist assumes a position such as that of the editor-in-chief, she will not be effective, according to the description by some participants. Participant No. 13 describes the issue as follows: “Some newspapers and news websites may resort to giving a specific position to a woman, not in way of recognition that she is competent; rather, it is to claim that they do not discriminate by gender, and in this case, almost all powers are delegated to her male deputy or assistant”. Participants No. 4, 5, 1, and 12 supported that “discrimination against females is rooted, and does not become less pronounced or diminish even after very long years of experience”.

Discrimination in **health insurance and social security is another form**, in that despite the efforts of the General Corporation for Social Security to include everyone in it, some electronic news websites evade that, but the strange thing is that some of these sites pay social security for men, not for females, without an objective justification for that. This was done in a fraudulent way against the Social Security Law. This was supported by Participants No. 14, 13, and 7.

Some participants pointed to the problem of nepotism or connections and their role in influencing the decision maker in the press institution, whether it is a paper or an electronic news website, and that it affects the decisions of appointment first, then promotion and the selection of sectors and institutions covered by the journalist, and other matters. Participant No.10 sees that “connections are the basis that leads you to what you want, and if you possess a certain level of qualifications and you have connections, it can lead you to the top.

Other participants mentioned the discrimination that occurs because of gender and related to laws; for example, the male journalist receives a family allowance while the female does not get such privileges. This is not limited to this aspect; female journalists cannot obtain health insurance for their family members, unlike male journalists.

Regarding **the veil (Hijab),** Participant No. 1 indicated that there was no discrimination because of it in printed newspapers, and that the majority of electronic news websites prefer non-veiled journalists. Participant No. 7 indicated that there was discrimination against a veiled woman if she were appointed. Some of the participants also complained of discrimination against them because they wore the veil, and stated that it is “undesirable as perceived by the owners of some websites,” according to Participant No. 11. Sometimes the veiled journalist is stigmatized as being from the Muslim Brotherhood, even though the majority of veiled females do not belong to any religious political group. Participant No. 9 indicates that some male journalist colleagues at the electronic news websites mock her for her prayer and hijab.

### Security, family and community constraints

**Political party affiliation** was mentioned by Participant No.1 referred to some harassment she was exposed to because she was working in an opposition newspaper affiliated with the Muslim Brotherhood. Harassment was manifested in the form of the difficulty of obtaining information from official authorities and being treated differently from other colleagues working in institutions known to adopt government views. Forms of this discriminatory treatment include not inviting the newspaper to attend official events, and delaying providing the press releases issued by official authorities. Furthermore, as a result of her previous work in a party-oriented newspaper, she still suffers from an inability to find a job, despite applying to jobs offered by many entities, including media and non-media organizations, whether these institutions are governmental or private. Participant No. 1 stated, “As a result of working in a partisan newspaper, a family member, such as a father, brother, or husband, may be exposed to pressure from certain parties to discourage the female journalist from working in the newspaper, especially if this journalist deals with sensitive issues.” The journalist added that the existence and continuation of the newspaper itself is unstable because of the newspaper’s partisan affiliation, and thus the high ceiling of expression in it compared to other non-partisan newspapers, whether daily or weekly.

Regarding **Security Harassment** participants noted that security harassment arises when the press institution is not aligned with the government but diminishes when newspapers align closely with the government, sharing its approach..Participant No. 3 denied being subjected to any security harassment during her work, which was supported by some other participants, while other participants refused it. Most of the participants attributed the matter to the nature of the journalist’s work, the content she presents, and its relationship to what the government wants or does not want so that the harassment increases whenever the content contradicts the government’s directions.

Sometimes, harassment and threats come from parties that the participants do not know, as indicated by Participant No. 10. Citizens or institutions that feel threatened by the journalist have an interest in harassing her or making a telephone threat. Participant No. 6 indicated that writing about a grievance against a security agency or a sensitive political topic may endanger journalists or electronic news websites.

At other times, the female journalist may be exposed to danger from a gang or the like, as happened with Participant No. 8 when security protection was imposed on her because she wrote against a gang and she had to be literally protected by security.

Regarding **challenges related to the family, parents, and relatives,** the participants pointed out several challenges. Participant No. 9 said, “My husband is my number one supporter, and without him, I would not have been able to work, and this led to him being stigmatized as weak and not having the ability to control me.” Female journalists from regions outside the capital faced initial resistance from their families, particularly from their brothers, who initially prevented them from working in the city, as noted by Participants No. 6, 9, and 15.

Despite parental support, female journalists struggle with exhaustion and face several challenges. Balancing household duties and childcare often works late into the night to manage both professional and domestic responsibilities. Participant No.13 noted that male journalists do not face this burden, as they are not typically responsible for household chores like their female counterparts do. This led to single female journalists refusing to marry due to fear of losing the job or for the prospective husband not accepting a job in journalism, or the inability to balance the burdens at home and work.

Participant No. 7 had a bitter experience where a person asked her to marry and stipulated that she quit work, and he said to her in one letter, “I am not a sheep or goat to let my wife work in the media”.

The researchers observe that female journalists from regions outside the capital encounter additional challenges beyond cultural differences. There is heightened conservatism in other cities, leading some female journalists to avoid marriage due to concerns about potential conflicts with their career or societal expectations placed on women.

The availability of a nursery at work, providing nurseries for children and allowing her to breastfeed her infant. Was an issue for recognizing their basic needs as mothers. Participant No. 5 said that “the institution I work in does not have a nursery, I have to take a leave so I can go to my mother’s house in order to breastfeed my baby; an arrangement which was made possible only because of the proximity of the work site to my mother’s house, and otherwise I would have resigned.” Lack of nurseries seems to be a problem for most working women, which affects their level of privacy and supports breastfeeding, possibly due to gender lack of equality in the workplace.

Discussions with the participants revealed the existence of **cultural beliefs** among many groups in society that affect the work of the female journalist and sometimes limit her ambition and ability to work. Participant No. 13 said that up to half of the male journalists did not accept the idea of a female journalist covering an event and limited their field to men. This is in addition to women’s inferior perceptions. Participant No. 9 told of a case in which things went even further to the point that one of the male journalists proposed marriage to a female colleague with the condition of leaving her work. Participant No. 8 asserted that customs and traditions did not affect journalistic work. Conversely, Participant No. 2 offered a contrasting view, stating that during emergency incidents at night, parents might disapprove of their daughters covering news late because of adherence to customs and traditions. Participant No.14 indicated that her family refuses her travel in any way. In compliance with customs and traditions that prohibit women from spending the night outside their homes. Participant No. 8 mentioned that the mother, first of all, usually tells her daughter that her end (destiny) is to be a mother and a housewife. Among the beliefs of male journalists, according to Participant No. 12, is that the normal place for females is in the kitchen, and those who rebel against this situation look down on them.

## Discussion

This study discussion was guided by gender theories: biological theory, social construction theory, functional theory, and socialization theory, each of which offers a different perspective on the concept of gender. These theories are not necessarily in conflict, but rather complement each other to provide a more comprehensive and integrated picture of how gender is shaped and affects individuals and society [27].

The findings of this study reveal a great number of challenges faced by the Jordanian female journalists, which were organized around which are organized around the workplace level: harassment and newsroom safety; pay inequalities, organizational culture and leadership which leads to subthemes as bullying, framing female Journalists, and stigmatization, On the other hands challenges themes found at the level of the society and family security which emerged several subthemes as party affiliation, security harassment, and cultural beliefs, as well as family, parents and relatives perspectives. Taken together, these themes underscore the nature of gendered experiences in journalism and highlight how organizational structure, culture and society factors play an important role in shaping the professional realities. Interpreting these results through focusing on gender theories allows for a clearer understanding of how individual narratives connect to broader patterns of inequality. This theoretical guiding would ensure that the discussion goes towards more systemic challenges influencing women’s participation and advancement in the media sector.

Harassments was one of the important findings that was highlighted by the participants whether the verbal harassment in the workplace within press institutions, or the sexual harassment in field journalism outside the press institutions, particularly during coverage of events such as demonstrations and protests which this was supported and reported by a report ‘“It is not your profession” [28] which showed that female journalists in Yamen, Tunis, and West Bank usually practice harassment against female journalists because they are women. The same was observed in a press release from Jordan [29].

Harassment could be related to the fact that female journalists who are subjected to harassment refuse to acknowledge the phenomenon and try to simplify it and include it in the category of a normal matter because of the societal culture that often tends to put women in the position of accusation, as they see in the way they dress and speak, where they sit, or the way they make themselves appear as a motive behind the men in general harass her. Therefore, for women journalists to relieve their embarrassment and pay societal accusations, they tolerate various violations and attempts at harassment that they may be exposed to during their work in the field, and turn a blind eye to them to the point that some of them classify cases and incidents of harassment as a normal matter that is classified as one of the dangers of journalistic work, that was consistent with North study [30]. While the report (The Missing Perspectives of Women in News) [31] indicates that corruption and sexual harassment in some countries prevent women from continuing to work in journalism. While countries that have enshrined gender equality principles in media laws have made a difference in women’s advancement to varying degrees, these efforts alone have not been sufficient to achieve radical change. This is partly due to deep-rooted cultural norms—patriarchal, male-dominated—that extend into newsrooms and prevent the desired positive change.

Interviewee comments suggest that incidents of harassment appear to be less frequent in printed press institutions compared to other media sectors. This might be related to differences between institutions. In that some institutions had developed fixed policies, which, in turn, has contributed to a reduction in sexual harassment. In addition to other factor which is constant oversight of the institution through presence of surveillance cameras or security personnel. This is consistent with other studies from different cultural backgrounds [14,32,33,34].

This findings regarding harassment are consistent with the social construction theory, which emphasizes that gender is a social construct learned and acquired through continuous interaction with others and participation in social life at all levels. Individuals learn the roles, behaviours, and expectations associated with their gender through family, school, media, and religious and cultural institutions, which act as agents of socialization. Researchers believe this theory contributes to understanding how stereotypical roles and behaviours can impose restrictions on individuals, especially female journalists, limiting their freedom and ability to realize their full potential. It also highlights the importance of changing social norms and values that perpetuate gender inequality between female and male Jordanian journalists and hinder the achievement of social justice and societal development [35].

Pay and promotion inequalities, were among the important findings in our study as these could be related to discrepancies not only restricted to female journalism; rather, they may reflect broader gender‑based discrimination across multiple sectors [36]. However the participants highlighted that discrepancies in wages and promotion were clearer in private media sectors that national one’s.These findings align with those of a study by Injaz [14], but contradict the conclusions drawn by Mobaideen [13] which showed that Jordanian female journalists expressed a high degree of job satisfaction.

The study findings related to wages and promotions contradict the functionalist theory, which holds that sexual differences or gender roles contribute to social integration and solidarity by assigning specific roles to each sex, contributing to the stability of the overall social system. This theory also holds that the gender division of labor is based on a partial biological basis, with both men and women performing the jobs for which they are believed to be better suited, biologically or socially. The researchers argue that this gender division of labor assigns higher-value roles to male journalists, and reproductive and nurturing roles to female journalists, reproducing conditions of men’s superior social status within the family and society, compared to women’s lower status. This, of course, is reflected in the differences in wage and promotion determinations between male and female journalists [35].

Bullying was among the challenges that the participants raised in the study. We believe that according to the prevailing traditions in Jordanian society, women’s abilities are viewed as less than men’s, and that men have power and authority in terms of culturally defined gender roles. This is reflected in the conviction of some male journalists working in the field of journalism that a woman’s place is in the home and raising children. Perhaps the acceptance of female journalists in press institutions for such bullying is due to the need to work, which forces them to remain silent about some practices that are offensive to them. This finding is similar to those of other studies [37–39].

Nawal El-Saadawi (the Egyptian writer and novelist who defends human rights in general, and women’s rights in particular) points out that the wife’s work outside the home is viewed by many Arab men as an insult to the man’s manhood. Masculinity, especially the man (the hero or the strong), requires that he be able to support his wife and not allow her to mix with men in offices, streets, or public transportation [40]. While Fatima Mernissi (Moroccan writer and social researcher, specializing in women’s affairs) believes that the problem of some modern Muslims with women’s rights has nothing to do with religion or Islamic heritage, but rather because those rights conflict with the interests of the male elite [41].

Our findings indicate that female journalists’ success is often stigmatized by male colleagues, who frame their achievements with suspicion—reflecting broader resistance to women’s advancement in media institutions. This aligns with Fatima Mernissi’s view that men’s fear of women leads them to confine and limit women’s influence [41]. Stigmatization could be related to the resistance of some male journalists to the idea of the female assuming her rightful status. The female’s ability over the past period to prove herself and deliver achievements made them sensitive to her. Furthermore, some male journalists come from cultural backgrounds that treat females as having a lower status than men, and this may be reflected in their view of them in the work environment and reduce their acceptance of their success. This leads to stigmatizing and framing of females with negative attributes, and this is consistent with the study by Adam [32] which highlight the importance of gender role. However this contradicts with the study [13], who highlighted that the estimates of Jordanian female journalists levels of obstacles faced by journalists in their work were low across all fields. Moreover, the findings reveal that female journalists face multiple forms of discrimination including pay gaps, limited promotions, restricted assignments, tribal affiliation, wearing Hijab and stigmatization reflecting entrenched patriarchal norms that undermine their professional status and opportunities. This finding is consistent with the report (Gender and media: Commitments of media institutions to gender equality) [42], which indicates that female journalists adapt to male-biased default professional standards and “masculine” values in order to integrate into the newsroom and advance in media institutions, or because they are not aware of an alternative way of working, or because this reality is imposed and difficult to change due to existing power dynamics. In addition to what Fatima Mernissi pointed out, who believes that the hijab is a symbol of unjust male authority over women [41]. Moreover, Forms of discrimination mentioned by the participants might be attributed to the gender role and gender issues within the overall institution. This is consistent with the findings of previous studies [12,14,34].

The word “discrimination” was repeated many times by the participants, and there was a general feeling and agreement on the existence of discrimination in different forms and in varying proportions, especially concerning wages, promotions, and assignments. This can be attributed to the recent and strong entry of females into the media field and their increased participation in electronic news websites. Historically, the full administrative control was for males, and with the beginning of females’ affirmation of their position/status and media-related capabilities, there was a kind of resistance among some, and an expression of reluctance to accept that females take positions and assignments greater than before. However, there are some deeply held beliefs in the minds of some, such as that the assignments to cover important places should be shared of males and that females cannot cover sovereign institutions. However, according to the opinions of the participants, females continue to prove themselves and gain advanced positions in some media institutions regardless of gender discrimination.

Our study revealed findings regarding bullying, stereotyping, stigmatization and various forms of discrimination against female journalists are consistent with the socialization theory [35], which considers socialization a crucial and fundamental process in shaping individuals’ gender identities from birth through adulthood and beyond. Through ongoing interaction with family, school, media, and other institutions, children learn the roles, behaviours, and expectations associated with their socially defined gender. This process is not merely the acquisition of information or simple imitation, but rather a deep internalization of the values and norms that society sets for each gender, shaping the core of their personality and their perception of themselves and their place in the world. This is evident from the practices of male journalists towards their female colleagues, as these negative practices of bullying and belittling them are a reflection of the standards set by society and on which these male journalists were raised.

Other study findings indicate that female journalists face political and security-related harassment, particularly when working in opposition or non-government-aligned newspapers, with discrimination ranging from restricted access to information and exclusion from official events to direct threats from authorities, citizens, or criminal group which underscores how partisan affiliation and sensitive reporting can heighten vulnerability in the media environment.This is consistent with other study [15].The researchers attribute security harassment to newspapers publishing press material that contradicts or differs from the journalistic orientation seen by the government and security services, which may be justified at times and unjustified at other times. The harassment and threats that male and female journalists are exposed to from other parties are due to journalists shedding light on issues that their owners want to hide and remain silent about, which is largely evident through inspective journalistic investigations. This is consistent with the United Nations Human Rights Report [43].

The findings highlight how family expectations, cultural traditions, and workplace structures intersect to constrain female journalists’ opportunities, reinforcing socially constructed gender roles, yet also reveal moments of resistance where women challenge these norms and become sources of pride within their communities. This is in particular consistent with Al-Saadawi [40] who indicated that the Arab countries believe that women were originally created to play the role of women and wives. In terms of serving at home and raising children. She works outside the home on the condition that she returns home to perform her basic duties toward her husband, family, and children.

Cultural believes and social restrictions, whether held by spouses, families, or the communities surrounding female journalists, consistent with the biological theory, which holds that biological makeup is primarily and fundamentally responsible for the innate differences in the behavior of men and women. The theory also suggests that men are supposedly superior to women in physical endurance by virtue of their biological makeup. According to this theory, women are viewed as bodies with a fragile physiological structure, unable to compete with the male body in various fields, especially those related to hard work that requires great physical strength. Their physiological structure enables them to relate to the emotional and nurturing aspects, especially those related to raising and caring for children. According to this theory, biological differences are the basis for shaping sexual identity and the social roles derived from it. Based on this theory, the majority of male journalists believe that journalism is a demanding profession with no fixed location or timeframe, and therefore they prefer to marry a woman who does not work in journalism. If they do wish to marry a female journalist, they require her to leave her journalistic career as a prerequisite for the marriage, a sentiment echoed by the majority of unmarried female journalists participating in this study. These findings are consistent also with the theories of social construction and socialization, which indicate that individuals learn the roles, behaviors, and expectations associated with their gender through interactions with family, school, and surrounding institutions. From an early age, individuals begin to learn the roles, behaviors, and expectations associated with their socially defined gender, which shapes the core of their personality, their perception of themselves, and their place in that society [35]. These two theories, which downplay the work and effort of female journalists while valuing that of male journalists, explain the perceptions female journalists have expressed regarding how male journalists view and treat them in the workplace, including the managers of the press institutions where they work, and their exclusion from many privileges granted only to their male colleagues. These theories also explain societal views and the restrictions placed upon women in various ways, suggesting that these views, both within and outside the press institutions, are shaped by upbringing at home and subsequently by various societal institutions.

The authors believe that some environments in Jordanian society still view journalism with scepticism as an acceptable profession for females, although other environments have become proud of the presence of female journalists in them. This is what some of the participants have about having become a source of pride for their families. Nevertheless, certain beliefs prevalent among some individuals may lead them to oppose the involvement of their female relatives in journalism. There might be pressure exerted on female relatives working in this field, discouraging them from working late or attending specific locations to cover news. There is also the fear of addressing certain topics to avoid being subject to particular pressures. However, many female journalists have proved the opposite. This is consistent with the [14,15] studies.

### Study limitations

The study faced several limitations, namely: The small number of female journalists registered with the Jordanian Press Association, compared to the number of male journalists. Female journalists’ willingness to discuss topics that are embarrassing to them in various aspects, such as harassment, threats, unequal pay, promotion, etc. Female journalists’ preoccupation with numerous work tasks and insufficient time to interview them, as several interviews have been postponed several times. A number of female journalists declined to conduct interviews due to a lack of time, particularly for married women. This was due to their preoccupation with completing work tasks and fulfilling their family obligations, such as social events for example. They were also preoccupied with family duties, such as bringing children home from school, teaching them, cooking, and taking care of the home, children, and husband.

### Recommendations

Based on the study results, it is recommended that special attention be paid to female journalists by setting policies and laws that support equal wages and promotions, harassment protection in news rooms and the press institutions in general, fair work opportunities that are transparent and based on work experience and qualifications. The study also recommends that the Jordanian Press Association ensure the promotion of gender equality in public and private press institutions, and support the maternity rights of female journalists by providing nurseries in the workplace and granting them fair maternity leave, regardless of the institutions in which they work.

Further research is needed to assess whether the work environment is generally attractive or repulsive to Jordanian women. Examining the perspectives of male journalists, newsroom leaders, and policymakers, as well as exploring the impact of digital media environments on gendered experiences. In addition to research on effectiveness of policies aimed at supporting gender equality and comprehensive social justice. As well as conducting broader and deeper research to clarify the difference between the challenges faced by female journalists working in government media institutions, and female journalists working in private media institutions.

## Conclusion

In conclusion, this study provides an in‑depth exploration of the multidimensional challenges faced by Jordanian female journalists across workplace, field, family, and societal domains. The findings reveal how deeply embedded institutional structures and cultural norms could shape women’s experiences in journalism. Female journalists continue to encounter harassment, unequal pay, limited promotion opportunities, and discriminatory organizational cultures that undermine their professional growth. These challenges are further compounded by societal expectations, family pressures, and cultural beliefs that restrict women’s mobility, autonomy, and perceived legitimacy within the profession.

The findings highlight how family expectations, cultural traditions, and workplace structures intersect to constrain female journalists’ opportunities, reinforcing socially constructed gender roles, yet also reveal moments of resistance where women challenge these norms and become sources of pride within their communities.

## Supporting information

S1 File Inclusivity-in-global-research-questionnaire. (DOCX)
